# Skin Diseases, Their Description, Pathology, Diagnosis and Treatment

**Published:** 1871-12

**Authors:** 


*Skin Diseases, their Description, Pathology, Diagnosis, and Treatment.* By Tilbury Fox, M. D., Lond., M. R. C. P. First American from last London edition. Edited by M. H. Henry, M. D. New York: Wm. Wood & Co., 1871.

The editor of the American edition has not followed the fault so often practiced by translators and editors of foreign works, of modifying and changing the teachings of the author by copious notes. The author in the preface says: "The present work may be regarded as a second and condensed edition of my two former works (Vegetable Parasitic Diseases, and Skin Diseases in General) combined, rewritten and recast, so as to suit both practitioners and students."

The work comprises over three hundred pages of matter, including a Formulary for use in treatment of skin diseases, and a copious Glossarial index.

Some considerable portion of the work is occupied by the consideration of diseases which are mostly confined to warm or tropical climates, and would, therefore, not come under the observation of the majority of American practitioners.

As a work to be placed in the hand of the student wishing to gain a knowledge of skin diseases it is quite valuable, and will undoubtedly be of use to the general practitioner.

